# Supplementary material for: Label-Free Detection of CA19-9 Using a BSA/Graphene-Based Antifouling Electrochemical Immunosensor
Source: Sensors (Basel). 2023 Dec 8;23(24):9693. doi: 10.3390/s23249693 (PMC10748090; doi:10.3390/s23249693)
Supplement: Supplementary file 1 [file sensors-23-09693-s001.zip › sensors-2699006-supplementary.pdf]

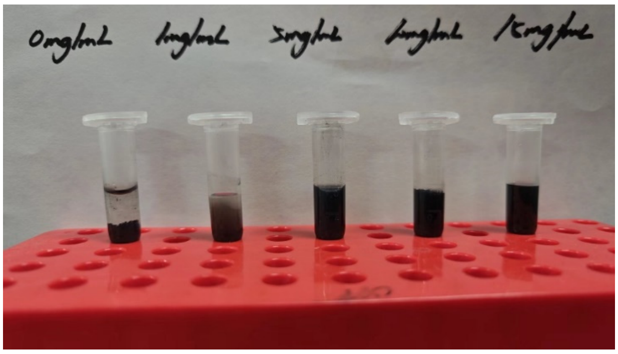

Figure S1. Comparation of BSA/Graphene nanocomposites with different BSA concentrations. The concentration of BSA in the samples are 0 mg/mL, 1 mg/mL, 5 mg/mL, 10 mg/mL, and 15 mg/mL, respectively.

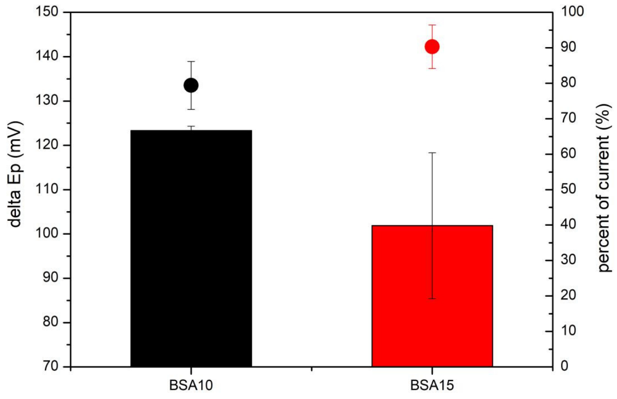

Figure S2. Statistical results of delta E_P and current change after BSA-Graphene-GA modification of AuEs. Columns denote delta E_p after modification, and dots denote the current percent.

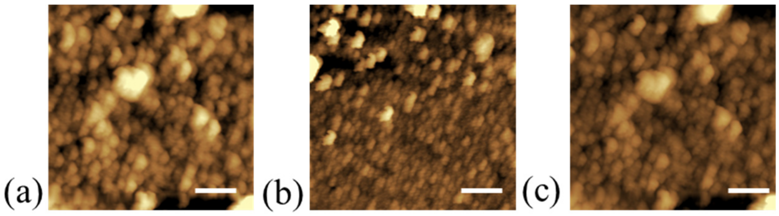

Figure S3. AFM images of BSA/Graphene/GA modified gold electrode (a), after anti-CA19-9 antibody capture on BSA/Graphene/GA modified gold electrode (b), and after antigen (100 U/mL) capture on BSA/Graphene/GA modified gold electrode (c). Scale bar 400 nm.

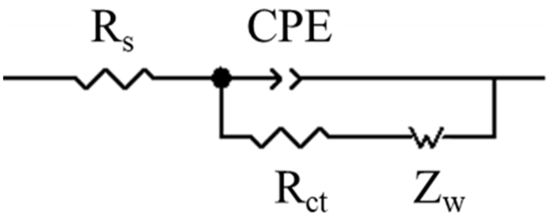

Figure S4. The modified Randles circuit model to fit the electrochemical impedance data.

Table S1. Comparison of this electrochemical immunosensor with previous reported biosensors for CA19-9 detection.

Electrode modification	Technique	Detection range (U/mL)	LOD (U/mL）	Ref.
GE/anti CA19-9/TiO2-SAb19-9-MB	DPV	1~100	1.6	[31]
Zn-Co-S/graphene	LSV	6.3~300	0.82	[32]
HPR–CA19-9/Au–CPE	EIS	2~30	1.37	[33]
CB-polyelectrolyte/anti CA19-9	DPV	0.01~40	0.07	[5]
PThi–SDS/AuNPs	EIS	5~400	0.45	[34]
MXene/HRP/anti CA19-9	DPV	0.002~30	0.001	[28]
BSA15/Graphene/GA/anti CA19-9	EIS	6.25~1000	13.5	This work
